# A theory-informed approach to identify barriers to utilising Point-of-Care Ultrasound (POCUS) in practice: from vicious cycles to sustainable solutions

**DOI:** 10.1007/s10459-025-10447-2

**Published:** 2025-06-23

**Authors:** Riikka Hofmann, Lenka Janik Blaskova, Nicola Jones

**Affiliations:** 1 Faculty of Education, University of Cambridge, Cambridge, UK; 2 School of Education, University of Exeter, Exeter, UK; 3 Institute of Applied Psychology, Faculty of Social and Economic Sciences, Comenius University Bratislava, Bratislava, Slovakia; 4 Royal Papworth Hospital, Cambridge, UK

**Keywords:** Point-of-care ultrasound, Clinical utilisation, Continuing professional development, Health professions education, Focused ultrasound training

## Abstract

Point-of-care ultrasound (POCUS) is a vital tool for diagnosis of life-threatening conditions, with broad consensus supporting its integration into medical curricula. Despite evidence of effectiveness of POCUS training, many clinicians do not utilise skills in practice, resulting in missed patient benefits. Research on the barriers to POCUS utilisation remains limited. To address this, we conducted a theory-informed exploratory qualitative case study to investigate the utilisation of Focused Intensive Care Echo (FICE) in a specialist heart and lung hospital. The investigation was framed using situated learning and activity theory. We undertook 28 interviews, three focus groups (*N* = 27) and two expert discussions. Thematic analysis identified barriers while difference-within-similarity-analysis (Hofmann, 2020) uncovered how these interact to hinder POCUS-utilisation. We demonstrate how barriers preventing trainees from using POCUS interacted with the wider activity system, forming vicious cycles to further hinder use. These vicious cycles related to enthusiasm, opportunity, support, participation, communication and norms that hindered POCUS-use, and manifest as an underlying tension between competing priorities of POCUS training and patient care. We discuss how theoretically re-framing the findings suggests low/medium-resource mechanisms which helped mitigate this tension and overcome the vicious cycles. These facilitative mechanisms could generate scalable and sustainable solutions to support POCUS-training and utilisation.

## Introduction

Advances in technology have made ultrasound devices more portable than ever (Baribeau et al., 2020) and Point-of-Care Ultrasound (POCUS) has emerged as a powerful tool to enable rapid, non-invasive diagnosis of life-threatening pathology at the bedside (Ghallab et al., 2023). Performed by treating clinicians rather than imaging specialists, POCUS can be undertaken immediately, integrating information directly into patient management (Oto et al., 2024). Given POCUS’s potential to improve patient outcomes there is widespread consensus that ultrasound should be incorporated into undergraduate medical education (Hoppmann et al., 2022) and postgraduate training curricula, for example in Emergency (Royal College of Emergency Medicine, 2021), Respiratory (Joint Royal College of Physicians Training Board, 2022b) and Acute Internal Medicine (Joint Royal College of Physicians Training Board, 2022a; Probyn & Daneshvar, 2025). Numerous POCUS-imaging protocols been developed for specific clinical scenarios with associated training pathways that develop skills and competence in image acquisition, interpretation, and clinical integration (Haney et al., 2020), some leading to accreditation.

Evidence is mounting that even relatively short POCUS-learning interventions are effective (Gibson et al., 2020; Millington et al., 2017; Vignon et al., 2018), particularly for image acquisition, but more rigorous training is required to become competent in image interpretation and clinical integration (Godement et al., 2019; Lenk et al., 2019; Millington et al., 2017; Wong et al., 2019). Once accredited, clinicians must continue practising to maintain POCUS-competence (Wong et al., 2019): skills decline is noted after just 1–3 months (Arnold et al., 2023; Gibson et al., 2020; Schnittke & Damewood, 2019; Yamamoto et al., 2018). However, despite POCUS-accreditation, many clinicians do not go on to utilise these skills in practice (Haney et al., 2020; Lien et al., 2022; Schnittke & Damewood, 2019). This represents significant resource-waste and fails to deliver improvements in patient care.

Limited research exists on the barriers to POCUS-utilisation in clinical practice after training (Haney et al., 2020; Lien et al., 2022; Wong et al., 2019); extant studies have focused on the technology, e.g. availability of ultrasound machines (Nanjayya et al., 2019), and the individual practitioners, e.g. lack of expert support (Bashir et al., 2021; Flower et al., 2020; Schnittke & Damewood, 2019). This resonates, however, with the wider observation that research on medical continuous professional development (CPD) consistently finds little evidence of change in practice or improvement in patient outcomes after educational interventions (Allen et al., 2021; Samuel et al., 2021). While not POCUS-specific, the CPD-literature indicates some consistently reported explanations as to why new guidelines/recommendations are not incorporated into practice; those may offer relevant starting points for framing and focusing an exploration of barriers to POCUS-use. This literature highlights the role of professional and interprofessional communication, workplace norms, opportunities for learning in communities of practice, access to feedback and clinical decision-making tools (Al-Omary et al., 2024; Allen et al., 2020; Forsetlund et al., 2021; Hofmann, 2024; Lau et al., 2015; Mather et al., 2022). What this literature further emphasises is that utilisation of clinical investigations, such as POCUS, in practice requires significant behavioural change, and calls for attention to the wider barriers, using a theory-driven approach (Lu et al., 2022; Steinert, 2020; Wong et al., 2020).

To explore these factors, we conducted a theory-informed case study. Given the insights from the CPD-literature that practice change involves complex professional learning, and depends on the institutional cultures and practices, we propose that professional and organisational learning theory (Lu et al., 2022; Wong et al., 2020), examining how individuals and organisations develop skills and apply knowledge to improve practice and embrace change, offers a useful lens to explore the barriers to utilisation of POCUS in practice and helps provide a more nuanced and multifaceted understanding of the factors involved. This might inform future training and lead to a more sustainable POCUS-utilisation model, enabling better resource-use and improvement in patient outcomes. More specifically, the CPD-literature indicated that implementation of new skills is not only a matter of individual’s knowledge/skill acquisition or CPD-training quality; it suggested that relational and normative features of clinical workplace practice, and access to feedback and tools within professional communities of practice may be important for implementation of new practices more generally. Theoretical frameworks that have conceptualised these aspects of professional learning have been under-utilised in attempts to understand translation of new individual knowledge/skill into clinical practice-use (Allen et al., 2020; Hofmann et al., 2024; Sehlbach & Pizzuti, 2024). We, therefore, propose framing our theory-informed inquiry through sociocultural theories of professional and organisational learning – notably situated learning theory’s notion of legitimate peripheral participation (LPP)^1^ (Lave & Wenger, 1991) and cultural-historical activity theory’s (CHAT) notion of activity systems^2^ (Engeström & Pyörälä, 2021) which highlight the situated, collaborative and tool-mediated nature of professional learning. These theories can help ‘make the invisible visible’ (Gormley et al., 2020), and are helpful for delving deeper into the nature of these barriers and how they could be overcome.

Being able to legitimately participate peripherally – before developing full expertise to participate centrally - enhances trainee doctors’ learning in and from practice (Allen et al., 2020; Buckley et al., 2019; Cruess et al., 2018; Hodges & Kuper, 2012; Steinert, 2020). Studies on clerks and residents found that opportunities for gradual exposure to (Chang et al., 2020) and participation in (Cho et al., 2024) authentic interactions generated confident learning and engagement. So did being taken seriously (seen and heard) and being supported to take responsibility (Adema et al., 2019; Chiel et al., 2022). Conversely, expecting too much knowledge/skill led to trainees’ withdrawal from engagement (Cho et al., 2024). LPP entails that student/trainee doctors are able to access to the artefacts/tools (Wenger, 2010) used in expert clinical reasoning and decision-making (Cho et al., 2024). These involve the primary material tools (e.g., ultrasound equipment, images; recording systems). They also involve ‘secondary’, non-material, tools expert doctors use in their clinical practice (such as diagnostic questioning and the thinking tools/representations of practice to integrate findings into clinical decision-making) (Battista, 2015). LPP highlights that these tools need to be articulated in clinical use-contexts, rather than relegated to courses/classrooms (Cho et al., 2024); trainees need opportunities to explain, and hear expert-clinicians explain, their reasoning (Chiel et al., 2022).

Clinical practices are embedded within the wider institutional activity systems: the workplace community with its norms and divisions of labour which influence how individual practitioners carry out their work (Hofmann, 2024). Explicit and implicit norms regulating participants’ actions and interactions in, and expectations of, their practice govern both to how to use the primary tools - ultrasound - and how to prioritise organisational goals (Battista, 2015) and can be hard to change. Within healthcare institutions, activity systems pursue more than one collective goal for the activity. Competing goals can generate tensions within healthcare institutions. For example, in many healthcare systems, the goals of caring for patients, on the one hand, and rationalisation of care, on the other, compete for time and resources. (Engeström & Pyörälä, 2021). Such competition may impact what practices are utilised and supported.

These theoretical insights guided our exploration of the barriers to utilising ultrasound in clinical practice. This study addresses three interrelated gaps in our knowledge/practice: the problem of lack of uptake of POCUS post-training, shortage of evidence to explain the lack of POCUS uptake, and a limited application of professional learning theories focused on the sociocultural aspects of practice change in studying the barriers to implementation of new practices after CPD and how to overcome them. Considering individual (perceptions of competence, confidence), interpersonal (support, discussion of tool-use, participation) and organisational (activity system goals, norms) factors, we answer the following:

1. *What are the barriers to utilising POCUS in practice after embarking on training?*
2. *How and why do these barriers hinder the utilisation of POCUS in practice?*

Deeper understanding of barriers to POCUS-utilisation may inform future training and lead to a more sustainable model for POCUS-utilisation, resulting in more effective use of resources and improvement in patient outcomes.

## Materials and methods

### Study design and settings

An in-depth, qualitative exploratory enquiry best supported our aims to understand the nature of barriers to POCUS-utilisation (Silverman, 2021). Gaining clinicians and practitioners’ perspectives helped understand how they experience POCUS in their settings. The study was located in a specialist UK National Health Service, NHS, (public) heart and lung hospital in the East of England. Two criteria made this location relevant for wider insight. Firstly, this hospital was an early adopter of POCUS-training, with a supportive institutional environment and presence of POCUS-experts for mentoring trainees in POCUS. It hereby present an ‘ideal-typical’ case to study (LeCompte & Goetz, 1982; Silverman, 2021): ‘typical’ in the sense that it was a non-selective and resource-limited publicly-funded healthcare institution; ‘ideal’ in the sense that there was institutional endorsement of POCUS-use and POCUS-experts were present in the setting. Ideal-typical cases enable the identification of ‘ambitious but realistic’ (Hofmann, 2024) practices: practices that could be feasible in other public hospitals in similarly-resourced healthcare systems, however, as this setting is ‘ideally’ placed to support POCUS-use while otherwise ‘typical’ in a general sense, if the participants in this setting are found to struggle, then other – both typical and ideal-typical - settings are likely to struggle too. The results will hereby highlight the kinds of norms and structures that would likely need to be in place/developed elsewhere to support POCUS-implementation.

Secondly, further testing of the findings in other settings came through the inclusion of current and past POCUS-accredited trainees from this hospital. Due to training rotation, participants had experience from a range of hospitals, including District General Hospitals; besides, some POCUS-participants had since become consultants in other hospitals. The dataset therefore enabled diverse institutional perspectives, including less ‘ideal’ settings, enhancing the wider relevance of its findings.

### The case

A specific POCUS-programme provided the case for this study: FICE was the first UK national POCUS-training programme. It focuses on point-of-care ultrasound assessment of the heart in patients that are haemodynamically unstable. Aligned with international POCUS-training pathways (Oto et al., 2024), training comprises three phases:

- Theoretical phase (online/in-person approved course).
- Mentored Logbook (collecting and documenting 50 scans under mentor guidance).
- Triggered Assessment (an experienced ultrasound practitioner directly observes the trainee scanning a patient).

Every trainee participating in this programme is allocated a mentor who is a clinician skilled in ultrasound to help with hands-on practice and logbook collection, and a supervisor with formal echo accreditation to undertake the triggered assessment. Participants need to complete this process within a 12-month period.

### Participants

To gain a multi-perspective understanding of barriers to POCUS-use, our study took a multi-professional approach to inviting participants: we included both trainees who had participated in FICE-training but were early in their FICE-use trajectory, and those who were already experts. Study participants included trainees, consultants, and other clinical professionals involved in FICE (see Table 1). We followed purposeful and snowball sampling to recruit participants with a range of experience in FICE from those beginning training to experienced practitioners. The sampling criteria are described in Table 1. We started recruiting participants from the 2014–2015 trainee lists of FICE-mentors based in our study settings. We invited participants to recommend further FICE-trained clinicians/practitioners to join our study. 28 professionals were interviewed individually and focus groups included 27 medics (trainees and experts) and healthcare practitioners, from anaesthesia, intensive care medicine, emergency medicine, cardiology, surgery as well as sonographers, cardiac physiologists, healthcare scientists and advanced nurse practitioners.

**Table 1:** Sampling criteria, supporting rationales and operationalisation

Aim	Purposive sampling criteria: Data needs to include	Rationale	Operationalisation of sampling criteria
Generating theoretical insights from qualitative research	Rich experiences and perspectives; A range of experience and perspective	To ensure that there are enough examples of the practice studied in the data so that we can understand it in-depth and that there is a sufficient range of examples to understand the different ways the barriers can play out	We included participants from/with experiences of different settings (e.g., as part of trainee rotation), including participants who had since the course moved to other settings (e.g., through rotation, or becoming a consultant)
Understanding the specific phenomenon: barriers to POCUS-use and how those might be overcome	Development of POCUS-use	To ensure we understand the nature of the phenomenon	We included experts and novices
	A multi-professional intervention	To ensure we understand the phenomenon in its real context	We included participants from a range of professions including doctors from different specialties (anaesthesia, intensive care medicine, emergency medicine, cardiology, surgery) sonographers, cardiac physiologists, healthcare scientists and advanced nurse practitioners

### Data collection

We conducted 20 main interviews in a 3-month period (2017). During preliminary analysis (2017-18), conducted by Author-1 and Author-2, we member checked our initial findings with two clinical FICE experts, Author-3 and another fully FICE-trained Consultant Anaesthetist. To reach data saturation, we collected further data through 3 focus groups, 4 additional interviews, and discussions during 2 echo-network workshops. These workshops - attended by Intensivists and Anaesthetists interested in POCUS and establishing a training programme - also served as opportunities for member checking through the involvement of clinical leaders. These methods supported an in-depth exploration of FICE-incorporation into practice. Collected data type and volume met key qualitative criteria for richness, relevance, and rigour as outlined by Larsson (2005):

- The data set and analysis contained both rich and consistent evidence for the barriers identified, and a nuanced understanding of those barriers (demonstrated by our identification of the way these barriers operate, and not just what they ‘are’);
- Collecting and analysing data from multiple professional groups involved in FICE-use enabled us to develop a better overview of the ways the barriers play out in inter-related ways, instead of simply listing a long list of individual barriers;
- Lastly, our rich multi-perspective dataset, informed by theory, enabled us to develop the conceptual understanding of the mechanism ‘vicious cycles’ in stopping POCUS-use, and identify solutions to overcome those.

The iterative development of the interview schedule (Appendix A) was informed by our theoretical constructs and strategies for qualitative interviewing (Kvale & Brinkmann, 2009). We used a semi-structured format, combining core questions with flexibility to explore emerging topics through prompts and probes (Kallio et al., 2016). Key focus areas included: (1) FICE training and/or skill sustaining, (2) work/ward culture, (3) barriers and enablers, and (4) best practice examples. A preliminary list of barriers, developed from 4 pilot interviews (2016), was shared with participants at the end of interview to expand reflection and discussion (cf., Hofmann, 2008). Phone/Video-call interviews helped access busy professionals across locations (mean duration = 26 min). Focus groups were conducted in-person (mean = 31 min). All data was collected by Author1 and Author2, audio-recorded and professionally transcribed.

### Data analysis

We analysed transcribed data in two phases of computer-assisted qualitative analyses to identify barriers (Phase 1) and understand how they hinder FICE-use (Phase 2). Phase 1 involved thematic analysis (Braun & Clarke, 2006), combining inductive and deductive approaches to identify barriers. First, we (Author2) coded data top-down to find barriers we (Authors1-3) had previously identified in the literature, theoretical framework, and pilot. This allowed us to thest the validity of the listed barriers by examining examples and surrounding data, exploring how these barriers manifested in practice (Silverman, 2021). Next, we (Authors1-2) followed a bottom-up approach to note new barriers missing in our list but mentioned by the participants. For each new barrier, remaining data was reviewed for related examples (Author2). Phase 1 analysis resulted in a list that included both previously identified and new barriers. The authors (Authors1-3) compared discrepancies (such as a different experience of the same practice factor or contrary examples to assumptions in literature/theory) to support the reliability and validity of findings, enriching the list without discarding any original barriers.

Phase 2 followed with difference-within-similarity analysis^3^ (Hofmann, 2020) to reveal how the identified barriers (Phase 1) prevent the adoption of FICE practice. We (Author1, checked by Authors2-3) analysed examples of the same barrier to practice and examined different perspectives on how it is being discussed as a barrier. This in-depth analysis resulted in more comprehensive understanding of the nature of the barriers and their connections to the wider clinical and normative context of practice. We validated our results via (1) word search (‘question’, ‘problem’) to identify further relevant data that may have been missed, and (2) repeated discussions and testing of the findings among the multi-professional team and with an expert panel on an echo network day.

Aligning with the Quality appraisal guidance of the National Institute for Health and Care Excellence (National Institute for Health and Care Excellence, 2012) ensured the rigor of our study. Findings were strengthened by triangulation of methods (interviews, focus groups), data sources (multiple participants in different professions and varied career levels), by regular discussions among the multidisciplinary research team (involving researchers trained in the learning sciences, clinical medicine and psychology) and member checks (focus groups, expert panel). In the Appendix B, we outline the multiple stages of using member checking in our study to enhance the credibility and trustworthiness of the study.

### Reflexivity

The research team was multi-disciplinary. Author-1 is a senior learning scientist and educational researcher with extensive expertise of theories and methods associated with studying professional and institutional learning, including in healthcare settings, based at an Education Faculty. Author-2 is a fully trained psychology and education researcher, based at Author-1’s faculty at the time of the study. Author-3 is a consultant in Cardiothoracic Anaesthesia and Intensive Care Medicine at the main institution of this study, with expertise in critical care echocardiography. To minimise bias while maximising contextual relevance, all the data collection was undertaken by Authors 1 and 2, who had no relationship with the hospitals in which the study participants worked/trained, or the participants themselves; nor did they have any role in the trainee-participants’ training or progression. Author-3, who was a consultant in the hospital in which all the participants worked/trained either concurrently or at some point in the past, was involved in the conceptualisation of the study, data analysis and write-up, but not data collection with participants. Author-3 did not have access to the dataset in a non-anonymised form; the initial data anonymisation, management and early stages of analysis were undertaken by Authors 1 and 2, however, Author-3 was involved in the later analysis and interpretation of the data, ensuring contextual accuracy and relevance of the interpretations for the healthcare setting.

## Results

The analysis identified a list of barriers (Table 2) which resonated with the literature. Further rounds of analysis suggested that identified barriers were not independent but inter-linked and form ‘*vicious cycles*’ that can become closed loops which stop trainees for developing their FICE-skills in their clinical settings. We identified 6 such vicious cycles which we now discuss, starting with individual trainees’ characteristics, moving to interpersonal perspectives and actions and onto departmental cultures.

**Table 2:** List of barriers

Aspect of Practice	Issues and concerns
Course itself and the ‘jump’ moving to real practice	Training artificial – not sufficiently linked with real use: Gap moving from training to independent use (‘Learning gap’)
Resources	Availability of scanners (number, cross-contamination issues when shared; infection control between patients) Time (competing demands)
Access to scanning	Resources (see above) Enough patients with a clinical need to allow opportunities to practice
Getting an image	Fear of the machine Difficulties getting image, particularly with Critical Care patients (e.g., drains, condition)
Type of patients	Type of patients (District Hospitals do not have critical mass of cardiac patients)
Interpreting an image and trainee confidence	Fear of interpretation (trainees) – confidence Fear of over-interpretation (seniors) – resistance
Availability of expert support	Lack of trained senior staff available to support; Lack of dedicated training time for (seniors) Consequences: Loss of momentum when no support; Loss of learning from errors (trainees)
Culture	Inter-professional barriers (surgeons don’t always see FICE as relevant/useful; Cardiologists may view as their area of expertise; Nurses can view as an unnecessary inconvenience to patient care) Senior staff not always supportive of trainee learning / Don’t see as priority / Culture of mistrust of junior doctors Absence of a general culture of acceptance of FICE
Risk	Clinical risks related to FICE Legal risks related to FICE Personal/Reputations risk to trainees in putting themselves forward

**Fig. 1:**
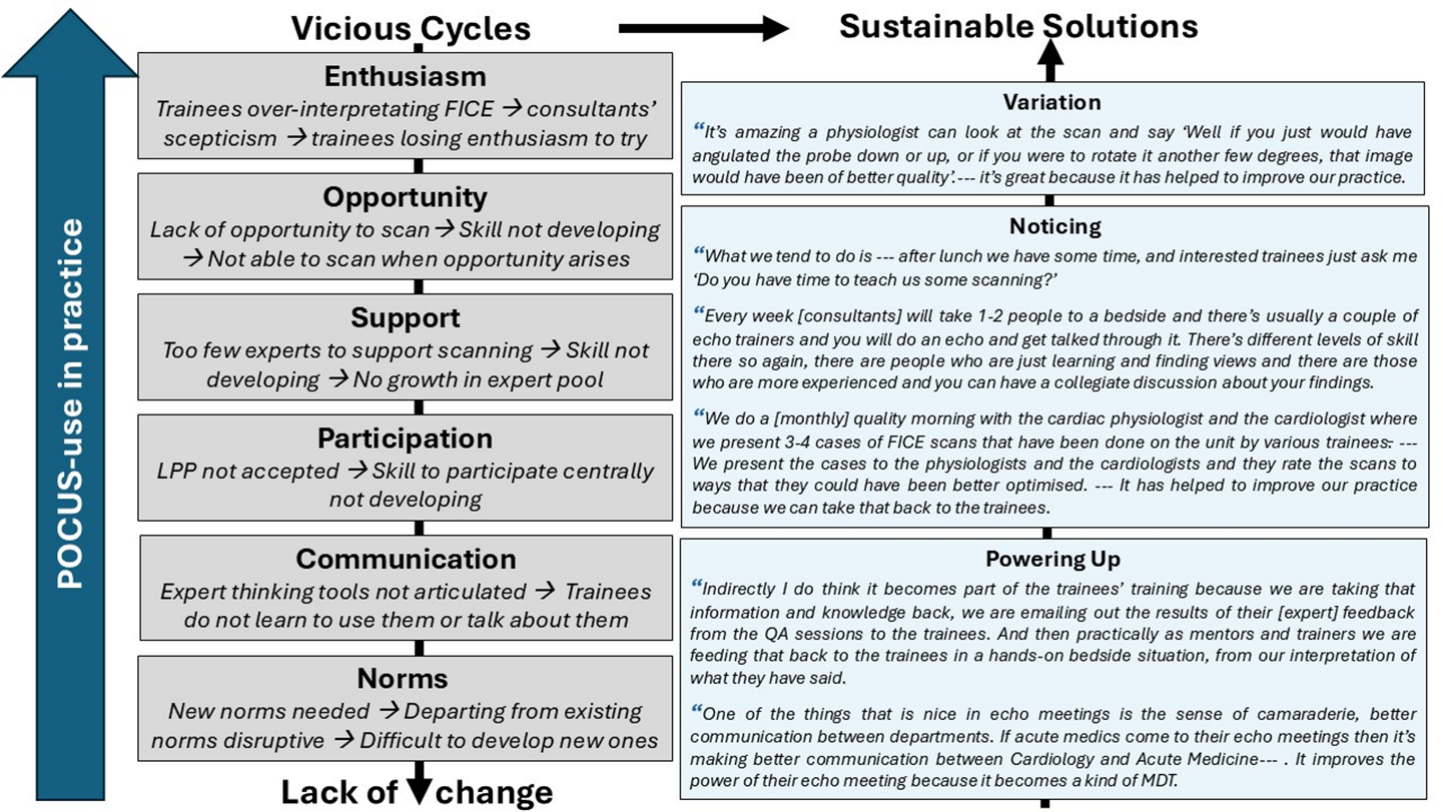
Solutions for opening up the vicious cycles that stop clinicians from using POCUS

An overview and summary of the six vicious cycles identified is provided on the left-hand side of Fig. 1.

### Vicious cycle: enthusiasm

The first vicious cycle we identified relates to trainees’ enthusiasm which is needed to get them off the ground using FICE, but can also lead to over-estimating their own skills. While trainees in our study were initially enthusiastic about using FICE after course completion, consultants expressed concern that some juniors are “absurdly trusting” of FICE (Consultant_General_M13) and “have maybe pushed the concept of FICE and what they’re really meant to be looking at, a little bit further than they should have been” (Consultant_General_M7). An advanced critical care practitioner concurred:

*I find with doctors [learning FICE] that they want to immediately start interpreting their scan --- rather than actually optimising their images. I always have to explain to them*,* ‘Let’s just go back to basics. I want you to be very good at giving me a good image before you tell me what you think about it*,*’ because I don’t feel it’s appropriate to start quantifying images that are still [sub]optimal. (ACCP_General_F7)*

Trainees themselves recognised that balancing enthusiasm with skill was challenging at the beginning:

*I think that was one of the hardest bits*,* learning how to use echo*,* but recognising that you are very much in the early stages and you really shouldn’t be making decisions on the basis of that. (Trainee_Specialist_M16)*

Consultants’ fear that some trainees are “interpreting things a little bit beyond what they should be” (Consultant_General_M7) generated resistance to FICE-use overall, through leading seniors to “lose faith in the whole system of FICE” (M1_1FG). FICE-sceptical consultants then dampened trainees’ enthusiasm: the focus groups suggest that over-interpretation can form a vicious cycle, whereby “surgeons only remember the wrong ones and not the right ones” (M1_1FG)”, getting “angry” and losing “faith” in FICE, with the consequence that trainees lose “the confidence to make the call” (M1_1FG). This raises the question how consultants and departments can foster/preserve trainee enthusiasm to learn FICE-use while avoiding safety risks.

### Vicious cycle: opportunity

Access to scanning to develop and sustain skills also posed a key barrier.

*One of the main barriers was --- the volume of scanning wasn’t that high in that unit*,* so people find it frustrating that they couldn’t complete their FICE-accreditation within the time limit. (Consultant_General_M5)*

Access can be limited by the volume of scanning (“there was a number of people trying to get the same scans on the same patients” (Consultant_General_M6). Besides,

*“The kind of patients that you have in the hospital make a difference*,* because [when] you have a lot of cardiac patients you can see a lot of different situations*,* and you can scan a lot of interesting cases and you can develop your skills. General hospitals who don’t have this critical mass of cardiac patients*,* it’s more likely when you scan a patient you find a normal heart*,* a normal finding and not finding pathological ones so it is more difficult to develop the skill and to become an expert.” (1FG).*

Consultants agreed that “one factor is the type of hospital --- if your hospital only operates on very healthy people that don’t require echo, then your training and your skills are going to be worse.” (Consultant_Specialist_M10). Yet,

*One of the problems with FICE is that it [the course] can be a bit artificial. --- the course kind of teaches you the very basic how do you do an echo*,* that’s it. It gives you a structure as to what your examination should be*,* broadly what you are looking for and then the mechanics of sticking a probe on a patient. And then it’s only once you start actually echo-ing real people who’ve got real problems that you start to develop the skills that you need. (Trainee_Specialist_M16)*

When access to practice is not available, then when a ‘real patient with real problems’ comes along, trainees cannot do it because they haven’t practiced, creating another vicious cycle.

However, we also found that while absence of suitable patients and protected educational time were factual barriers, we found supportive experts ‘noticed’ more clinical opportunities to scan a patient:

*There has to be some reason [to scan]*,* but that could be a fairly soft indication. (Consultant_General_M7)*

This kind of approach helped overcome the clinical need-vs-learning-need contradiction and we will return to it in the Discussion.

### Vicious cycle: availability of support

Even when ‘real patients with real problems’ are available, getting an image poses significant challenges: “Particularly in ICU patients who are ventilated --- and the technical difficulties so just getting an image is a big start.” (Trainee_Specialist_M16) A consultant confirms that particularly in ICU “it is one of the common difficulties” (Consultant_Specialist_M4). Trainees need expert support to deal with this.

*I kind of grew in confidence while I was there*,* and of course you have the advantage of having quite a few people with echo skills there so again there are people you can turn to and say ‘I don’t know but this looks to me like that’ and you can get immediate feedback. (Trainee_Specialist_M16)*

If expert support is not available, sustaining trainees’ enthusiasm is challenging,

*“because if you put the probe on the chest and you’ve got a rubbish image*,* and there is no one to tell you how you should correct that image --- then your enthusiasm for doing the scan in the first place very quickly disappears.” (M31FG)*.

Absence of support stops trainees from developing their skill of getting an image even where patients are available, because “if no one’s ever telling you you’re making mistakes you never learn from it --- you’ll carry on making that same mistake again and again.” (Consultant_General_M11) This continues to be a barrier beyond formal accreditation: “the problem with being the only one who’s FICE-accredited in the hospital, is that you cannot communicate with someone else who is FICE-accredited to just see if you’re on the same page and if you have the right opinion on the matter.” (Consultant_General_F2).

Lack of expert support can lead to a cycle where trainees lose the momentum with FICE. However, offering such expert support is often challenging because “if there are very few of those experts around basically it takes all their time” (F1_2FG).

This challenge is exacerbated by the activity system which does not prioritise training-in-practice:

*That’s the most difficult part because we don’t really have an education moment integrated into our working day*,* --- so indeed that’s a disadvantage. (Consultant_General_F2)*

The challenge for trainees is that in the absence of support (not as a one-off, but as a routine part of the practice), they often cannot begin to use FICE to care for patients.

*If you’re not training your people to do echo*,* you really can’t expect them to do it --- So you need that sort of supportive training environment*,* you need it being done routinely so people don’t think it’s esoteric and strange and you need even those consultants who are not echo-happy to be happy with the idea of doing echo. (Trainee_Specialist_M16)*

Limited numbers of experts to offer support and lack of dedicated training time restricts the support available to trainees meaning trainees’ skills do not develop; this creates a vicious cycle whereby trainees do not become experts, and hence the pool of experts who can offer support does not grow. However, support is not only about direct guidance, but about how novices can and are allowed to participate in the first place.

### Vicious cycle: peripheral participation

Trainees initially need to be able to participate peripherally, not independently. Above we discussed how trainees can lack “the confidence to make the call” (M1_1FG). Apart from “intimidation on seeing a foreign machine” (Consultant_General_F3), this involves “overcoming that fear --- what you think you see” (Consultant_General_F2). Focus groups confirmed that “it takes a long time for trainees to get to a level where they are confident enough to go and by themselves and put a probe on a chest and make a decision as to what they are seeing” (F1_2FG).

*You’re a little bit scared in the beginning and you’re definitely not sure about what you see*,* or what you think you see*,* just getting someone who has more experience and then overcoming that fear. (Consultant_General_F2)*

We suggest this is about opportunities for legitimate peripheral participation initially. The analysis suggests this was often not accessible, with consultants bypassing trainees in seeking scans.

*[The Consultant] did not ask us to perform the scan [they] just took the machine*,* the probe*,* and performed it on their own. --- I would prefer [the consultant] to take us and to show us and to do it with us. (Trainee_Specialist_M12)*

Peripheral participation is not always constructed as legitimate:

*If you see people who are FICE competent and who do it on a daily basis then it’s faster and quicker for them to get a quick picture of what’s going on*,* and then decide what they want to do next. Whereas if you’re not trained and if you’re trying to do it in an urgent fashion then it’s more time consuming and sometimes it just delays the whole process. (Consultant_General_M8)*

If trainees cannot legitimately participate in FICE peripherally initially, it is more difficult for them to develop their skills and confidence participate more centrally. Beside accepting peripheral participation, supportive cultures also need to be *educative*, employing effective articulation of strategies and problems.

### Vicious cycle: communication

Further to technical skills, clinical reasoning prevented trainees from gaining and interpreting images. Trainees asked limited questions to guide their thinking and decision-making during scanning, which made being ‘able’ to do it harder. Typically, trainees viewed FICE processes and related challenges as two key steps of (i) getting and (ii) interpreting an image.

*I’m still questioning in terms of; Am I getting the right images? Am I interpreting them correctly? (Trainee_General_M3)*

FICE-experts considered this 2-step FICE-representation insufficient, highlighting that FICE-use must start with a question.

*Before you do an echo you need to have a question. Unfortunately*,* most of my trainees right now think that it’s a good idea to do a scan*,* but they don’t know what they are looking for. And if you don’t know what you are looking for you will never find anything. (Consultant_Specialist_M2)*

And the results cannot be interpreted on their own. They require “integrating the information that you get from FICE with the clinical picture of the patient” [Consultant_Specialist_M4].

*What I don’t do and I think is hazardous if people just go and with unfocused view*,* perform an echo*,* form an echo opinion about the heart and then intervene on the basis of that*,* just that finding*,* I think is potentially quite risky. So I quite like to have some confirmatory clinical finding. (Consultant_General_M7)*

The trainees’ secondary tool, their representation of FICE-in-use, conflates with the primary tool, the material dimensions of FICE (the scanner, the probe and the actual image produced): it focuses on 2 steps, getting and interpreting an US-image. Whereas experts use 4-step model which expands and re-contextualises the primary tool: getting an image is preceded by a question as the first step, that the image is intended to help answer, and the interpretation is framed by the wider clinical context of the patient. However, there was no evidence that this gap was being widely and effectively communicated between the experts and the novices. Without explicit communication about the FICE-related reasoning gaps, trainees take much longer to develop their competence.

This suggests that ineffective communication across the professional groups may have limited trainees’ participation in full expert practice.

### Vicious cycle: norms

Lastly, workplace cultures are not about individual practitioners’ views/stances: workplace norms regulate who can do what, where, how, when within the activity system. Even where real patients are present in the clinical setting, practice, opportunities may not materialise because norms foresee that:

*You wouldn’t automatically do an echo on a patient. It’s a waste of time and it’s a waste of money. -- You have to do things led by clinical need. (Consultant_General_F5)*

Trainees report having been told by consultants that they ‘get annoyed with scanning for scanning’s sake’ [Trainee_Specialist_M14]. Norms regulating perceptions of hierarchy also impact on opportunities.

*I was an accredited TOE echo-cardiographer [but] --- because he was a consultant he didn’t quite believe me. (Consultant_General_M11).*

This sometimes links with more specific departmental norms about FICE-use, referred to in the interviews as a “culture factor even within the ICU consultants” which involves “the belief and buy in to the FICE and the FICE-training and the appropriateness of trainees doing scans on patients. Some consultants are very open to that and very willing for that to happen, and some consultants are less open and less willing for that.” (F1_1FG) “Departmental enthusiasm” is important as it is difficult to progress with learning and implementation if seniors in the setting do not “see the point of it” (Trainee_Specialist_M14). This may mean seniors do not allow “allow time for it, because they don’t see what the point is” (F3_1FG).

There also sometimes existed “a lack of clarity about who would be doing it”, for example when interprofessional cultures and norms regulate a setting “where cardiology isn’t interested in us having a look at the heart with an echo” (Trainee_Specialist_M14). This was confirmed by consultants: “to get a cardiologist as a barrier to echoing would stop more people from echoing” (Consultant_General_M5). Such interprofessional norms acted as barriers to FICE-use and learning.

*I think if you’re going to get a cardiologist to police your echoing you wouldn’t get very far. Because I’ve found it’s been antagonistic rather than facilitatory most of the time. (Consultant_General_M5)*

Such normative barriers were not restricted to cardiologists:

*In district general hospitals a lot of the ICU consultants can’t scan themselves*,* and don’t necessarily see the value of scanning. So it can then be difficult if you are an enthusiastic trainee in that hospital to try and progress your skills*,* because there is no one who can help you do that. Or can allow time for it*,* because they don’t see what the point is. (F3_1FG)*

Perceptions of surgeons were also described as a potential barrier.

*They [heart surgeons] are quite happy to have a cardiology registrar who may not have passed all the accreditation come and look at the patient*,* whereas they don’t believe the intensive care doctor --- So they kind of said*,* ‘Oh well*,* it’s nice that you did your scan but I want someone else to look at the patient.’ Fine. Okay. So there’s been a lot of scepticism from the non-intensive care people and the non-interested cardiologists. (Trainee_Specialist_M14)*

Using FICE without full expertise risks stepping on the toes of cardiologists, surgeons, nursing staff. Trainees reported that “heart surgeons prefer a cardiology registrar, they don’t believe in FICE-trained intensive care doctors” [Trainee_Specialist_M14], while consultants reported that “if you [as a trainee] want your numbers, to also [have to] make a deal with the nurse” – suggesting fitting with the norms of nurses’ work as a strategy, such as trainees could “say ‘If I come back at a certain time, what’s the best time to do a quick, you know, ten minute scan?” [Consultant_General_M6]. The vicious cycle of norms can emerge because norms are obligating to participants and hence where they limit opportunities to FICE-use and learning, new practices cannot emerge without changing those norms. Yet, departing from established norms is viewed negatively, making it challenging.

## Discussion

POCUS has emerged as a powerful tool in healthcare with potential to support sustainable improvements in patient care. This study addressed the problem that while POCUS is widely acknowledged as a core skill for doctors, particularly in the acute specialties, and US-training is well-received by students/trainees and supported by national training programmes/pathways, clinicians often do not incorporate US into clinical practice after training, leading to skills decay, resource waste and lost opportunities to benefit patients. The reasons for ineffective POCUS-utilisation are poorly understood and under-theorised. This study advances our knowledge of the barriers to incorporating POCUS into clinical practice, and of the use of sociocultural theories of professional learning to help identify ways of overcoming those implementation barriers. Hereby our study may also contribute wider understandings of the barriers to uptake of new knowledge/skills after educational interventions.

Conducting a theory-informed exploratory qualitative case study of FICE (Focused Intensive Care Echocardiography), we built on sociocultural professional and organisational learning theory, notably situated learning and cultural-historical activity theory (CHAT), to understand the nature of these barriers and how they might be overcome.

We identified a range of barriers that stopped trainee doctors from using FICE in their clinical practice after training, resonant with the literature on POCUS-use: over-interpretation; expert support (in CC: (Flower et al., 2020; Humblet et al., 2020; Jacques et al., 2017), emergency (Bashir et al., 2021; Haney et al., 2020; Schnittke & Damewood, 2019) and internal medicine (Wong et al., 2020); access to suitable patients (Arnold et al., 2023; Lien et al., 2022) and expert reasoning and quality assurance (Arnold et al., 2023; Bashir et al., 2021; Humblet et al., 2020; Wong et al., 2020) and departmental cultures (Flower et al., 2020; Gibson et al., 2020; Ginsburg et al., 2023), manifesting the lack of opportunities for legitimate peripheral participation (Lave & Wenger, 1991). Importantly, we showed how these barriers interacted with the wider activity system‘s goals, norms and divisions of labour (Engeström & Pyörälä, 2021), sometimes forming what we called ‘vicious cycles’: closed loops which hinder change towards POCUS-utilisation and learning and are hard for individual practitioners to overcome. Further building on sociocultural theories, we now discuss possible facilitative mechanisms that could help overcome these barriers without significant additional resource or wholesale organisational system change.

### Theoretically re-interpreting the findings and identifying possible solutions

Building on CHAT, we suggest that what underlies these challenges is a more fundamental tension between two activity systems acting in the hospital settings, patient care and capacity building of future doctors, with competing objectives, roles and norms of practice (depicted in Table 3). This underlying tension is difficult for practitioners to change, however, integrating our findings with the theoretical framework, we propose three potential facilitating mechanisms that may help mitigate the systemic challenges arising from this system(s)-contradiction to open up the identified vicious cycles; variation, noticing and ‘powering up’ support. Figure 1 illustrates these.

**Table 3:** Characteristics of and differences between two competing activity systems present in the studied setting

	Competing objectives of the practice	
	Patient care	Capacity building
Character of a priority	Clinical	Trainee learning, Professional development
Role of a trainee	Junior medic	Future consultant
Time perspective/ orientation	Now	Future
Risks	Immediate: Patient safety Medium-term: Legal	Medium-term: Resource waste (through training and opportunity costs if training does not lead to incorporation of POCUS in practice) Long-term: Lack of future capacity
Resources needed	Scanners; Infection control; Time (efficiency/ competing demands) Senior staff time advising juniors with scanning vs. patient time need	Scanners with available support (near or distant) AND Patients (availability, variability) Trained seniors Seniors’ time available to support
Rules / Conventions	Inter-professional boundaries	Inter-professional learning
Practitioners’ perspectives	Junior - Senior Inter-professional: Stepping on toes	Could reduce workload for cardiologists; Surgeons: could stop some patients from going into theatre unnecessarily
Community of Practice	Commonly the immediate community of professionals in the ward and the hospital	Cross-professional; Cross-departmental; Cross-organisational

### Variation

Availability of suitable patients limits *repetition* (i.e., more scanning) as a source of trainee learning. However, trainees recognised that the type of scans that matter more than their number: “[I] saw lots of normal scans but it is seeing more abnormal scans, you build up your bank of ‘this doesn’t look quite right’ type image” (Trainee_General_M3). This resonates with sociocultural theory’s suggestion that variation, rather than repetition, in learners’ observations/practice improves learning transfer as applying learning in new situations – such as new slightly different images – requires learners to adapt their knowledge through an understanding of the new situation (Fredholm et al., 2020; Marton, 2006). Trainees’ learning from physiologists suggests that access to greater *variation of images* need not rely on patients. We suggest that beside using ultrasound simulators with pathology (Dietrich et al., 2023), learning from variation in images/interpretation could potentially be achieved through developing/sharing image banks to cost-effectively enhance training.

### Noticing

Some consultants/departments were more sensitive to ‘noticing’ learning opportunities within existing systemic workflows and barriers. We suggest that noticing - the capacity to attune to certain aspects of professional practice to enhance meaningful learning opportunities (van Es & Sherin, 2021) – may be a key mechanism by which teaching hospital consultants can balance the competing tasks of training and patient care (Raia & Smith, 2020) within existing activity system norms/conventions. While absence of suitable patients and protected educational time are factual barriers in many settings, we found supportive experts ‘noticed’ more clinical opportunities to scan a patient (identifying ‘soft’ reasons to scan), helping overcome the clinical need-vs-learning-need contradiction. Similarly, some settings ‘noticed’ learning opportunities within existing flows/structures and built these into training routines.

We suggest that brief but regular teaching moments could help overcome the patient care-training contradiction; we observed examples such as:

- A lunch time education hour;
- ‘Echo rounds’ (a pre-set weekly session of an expert consultant going through the ward with trainees and a POCUS-device);
- Quality Assurance -meetings used as a trainee learning opportunity while simultaneously fulfilling other, mandatory purposes, was another example.

Beyond enhancing support availability, we identified an added benefit from these educational mini-routines, which we refer to, paraphrasing a participant, as ‘powering up’ support.

### ‘Powering up’

LLP should entail opportunities for trainees to discuss POCUS in authentic settings and to access experts’ thinking tools (Battista, 2015; Chiel et al., 2022; Cho et al., 2024; Haney et al., 2020). Quality Assurance-meetings provided scalable, authentic settings where trainees could observe multi-professional experts analyse and discuss images - learnings that, as our analysis suggests, transfer to bedside practice (see narrative quotations in Fig. 1). Additionally, utilising multi-professional meetings as learning spaces may support culture change by enhancing relations (‘camaraderie’) and mutual understanding and expertise across specialties/professionals.

Our study tentatively suggests that these facilitative mechanisms could help generate scalable and sustainable solutions to support POCUS-learning and use in clinical practice. In Table 4 we provide examples of the kinds of activities/resources that could operationalise these mechanisms in healthcare settings, and explore the associated resource implications.

**Table 4:** Scalable facilitating mechanisms to support POCUS-uptake, possible operationalisations with resource implications

Facilitating Mechanism	Description	Example / Vignette	Resource Implications
Variation	Variation, rather than repetition, in learners’ observations/practice improves learning transfer as applying learning in new situations – such as new slightly different images – requires learners to adapt their knowledge through an understanding of the new situation.	Clinical Practice	Low cost as happens in course of everyday practice; may be unpredictable so difficult to harness for systematic use but may be helpful as part of a wider set of strategies
		Image banks	More predictable; cost could be managed through aggregating images emerging from clinical practice and pooling of images with other providers; does not provide hands-on scanning experience
		Simulator	More predictable and provides hands-on experience but requires investment
Noticing	Noticing - the capacity to attune to certain aspects of professional practice to enhance meaningful learning opportunities [49] – may be a key mechanism by which teaching hospital consultants can balance the competing tasks of training and patient care [50] within existing activity system norms/conventions.	Ad hoc in practice: e.g., on ward rounds	Offers real patient exposure; low cost as happens within routine care; may be unpredictable so difficult to harness for systematic use but may be helpful as part of a wider set of strategies
		Specific teaching sessions e.g. ‘echo rounds’	Provide structured, supervised learning with feedback; availability may be limited by trainer capacity and clinical demands; building a culture where opportune moments are grabbed for brief ‘live’ sessions may help capitalise on available capacity.
		Formal multidisciplinary meetings e.g. ‘echo QA meetings’	Enables exposure to diverse cases and expert interpretation; cost may be managed through building on existing staff meetings; retrospective and lacks hands-on scanning.
Powering up	Legitimate Peripheral Participation provides opportunities to discuss POCUS in authentic settings to access experts’ thinking tools multi-professional experts discuss and analyse images	Informal interactions with experts	Timely and contextual learning, but unstructured and unpredictable; building a culture where opportune moments are grabbed for brief ‘live’ sessions may help capitalise on available capacity.
		External events (conferences, podcasts)	Broader insights and networking; can be costly and less interactive.
		Fellowships or extended placements	Deep learning and mentorship; time-intensive and limited access, not in itself scalable but may help build lead expertise to lead culture change.

### Limitations

Our study was conducted in a specific local context and generalising the findings requires caution. However, the resonance of the identified barriers with the literature from other settings, and the interpretation grounded in well-established professional and organisational learning theories relevant to healthcare contexts/development, suggest that our findings may have applicability across other hospital, national and specialty settings. We discuss this further below.

### Relevance and future research

#### Temporal relevance

The national Focused Intensive Care Echocardiography (FICE) training programme was introduced in 2012, with this study conducted during 2017–18. In 2020, FICE and the Critical Care Ultrasound (CUSIC) programme were merged to form the FUSIC (Focused Ultrasound in Intensive Care) accreditation framework, which has since expanded to include additional modules. Despite these developments, the core training structure—comprising e-learning, formal course, mentored practice, logbook completion, and triggered assessments—has remained largely unchanged. Trainer availability and ongoing clinical service pressures continue to represent significant contextual constraints. The findings of this study therefore remain highly relevant to understanding persistent challenges in the implementation of ultrasound training in critical care.

#### Relevance to other forms of POCUS

Although FICE is specifically intended for the assessment of cardiac function in haemodynamically unstable patients, it is broadly representative of point-of-care ultrasound (POCUS) practice more generally. Both involve bedside imaging performed by non-imaging specialists to address focused clinical questions in acutely unwell patients, with interpretation by the clinician directly involved in care. The training pathways are similarly structured, typically comprising e-learning, a formal course, mentored practice, logbook completion, and triggered assessments. The findings of this study therefore have broader relevance for POCUS training and continuing professional development.

#### Relevance to other types of hospitals and healthcare systems

As discussed in the Introduction, lack of POCUS-use after training is a poorly understood problem across countries. However, more general CPD-implementation evidence from different healthcare organisations and systems indicated that the identified hindering factors feeding into the vicious cycles - confidence; opportunity to utilise new skills; support/mentoring; access to peripheral participation, effective communication and institutional practice norms (Chiel et al., 2022; Cho et al., 2024; Lau et al., 2015; Witti et al., 2023) – are common barriers to uptake of new practices. This offers support to the potential relevance of our findings in other types of hospitals in high-income countries. In low-and-middle-income countries (LIMICs), access to US-machines and equipment are common key barriers (Ginsburg et al., 2023), precluding the use of the kinds of strategies we identified. However, also in LIMICS, when machines and training are available, similar barriers to ours have been identified (Ginsburg et al., 2023; van Hoving et al., 2022), suggesting our findings could be worth exploring further. We note that for rural settings, while access to expert mentoring and feedback/quality assurance have also been identified as barriers to POCUS-use (Arnold et al., 2023; Morton et al., 2024), the lack of teams of colleagues and suitable patients may make the applicability of our findings more limited.

#### Future research

Our discussion above suggests our findings may have relevance in other reasonably-sized (non-rural) typical and ideal-typical settings in HICs, and reasonably resourced LIMICs, where equipment and training is available. Their applicability across such settings facing challenges with post-training POCUS-use, therefore, warrants further research: our theoretically-informed nuanced yet structured findings can inform both in-depth qualitative and larger-scale quantitative research on the wider presence of these vicious cycles in hindering POCUS-use, and possibly other new practices, after educational interventions. Moreover, our study calls for further research on the potential scalable solutions identified in our study to overcome these barriers, including local/qualitative studies of feasibility and associated resource-needs, as well as larger-scale evaluation studies on the effectiveness and cost-effectiveness of these solutions for overcoming barriers to POCUS-use in scalable and sustainable ways.

## Conclusion

Our findings can help develop scalable ways of addressing of common barriers to POCUS-use after training and thereby ensure sustained benefits to patients and the taxpayer. Moreover, halting post-training skills decay could quickly build capacity, thereby benefitting future patients by creating virtuous cycles whereby an increasing population of capable and confident POCUS-users could better support trainees’ LPP and bring maximum patient benefits. Consistent with situated theories of professional and organisational learning, the barriers and facilitating mechanisms identified may also help shed light into the wider observed problem of translation of learning from medical CPD into clinical practice.

## Appendix A

**Interview schedule**

**Table:** 

	Question	Prompts
***A***	***FICE training and/or sustaining skills***	
A1	What is your current job title?	*If not*,* what is your most recent job title?*
A2	Which year did you do the FICE course? Including the logbook and triggered assessment?	
A4	How are you maintaining your skills?	*Have you attended further PD events about echocardiogram?* *How often do you attend these?* *What has enabled/hindered you to do so?* *Do you still scan? How many times/week/month on average?* *In what situations (workplace*,* training*,* arrest)* *For what uses? (Diagnosis*,* Follow-up*,* Monitoring*,* Screening)*
A5	Could you describe in detail a recent occasion when you did a scan?	*How typical was this occasion compared to other situations in which you scan?*
***B***	***Work/ward culture***	
B1	Is using echo typical/common in your work setting?	*Who uses it (all)? Why and how do they use it?* *Could it be more common (is there more need for it)? What could help make it more common?*
B2	If you have worked in different wards/settings, have you observed differences in echo use? Could you describe those?	*What do you think is the source of those differences? (Or if only one: What does that example tell about the culture on your ward?)*
B3	In your experience of scanning, which groups of people may sometimes influence your decision to scan or the scanning situation?	*Consultants? Peers? Junior doctors? Nursing staff? Patients/Relatives? How does that vary between settings?* *Do other staff in your setting ever have any concerns about using it when you suggest it? [Have you got an example?]*
***C***	***Barriers and supporting factors for using echo [some may not be needed after B and list]***	
C1	Have you got any concrete support from your organisation/department to use echo? From whom?	*Have you got an example?* *What was the impact on your practice?* *How about indirect support?*
C2	Have you got an example of when you used echo and it was a really positive experience? How often does that happen?	*What happened? Did it make a positive contribution to your clinical practice? What enabled that?* *Is that a common occurrence?*
C3	Have you got an example of when you wanted to use echo and then didn’t or it was difficult?	*What happened?*
C4	Have you got any comments on the FICE course?	
C5	What would help you use echo more efficiently?	

## Appendix B

**Data collection, member checking, and analysis outcomes**

This section complements the Data collection and Data analysis sections in the main paper.

**Table:** 

Phase	Data source	Objective	Collected by	Analysis Outcomes	Member check
0	Pilot interviews (*n* = 4)	Identify barriers and enablers	Author 1	1.Preliminary list of barriers and enablers; 2.Interview schedule 3.Early themes	Author 2, Interviewees
1	Interviews (*n* = 20)	Identify links and process between the identified barriers and enablers	Author 1, Author 2	1.List of barriers and enablers finalised to include original and any newly identified barriers. 2. Nuanced understanding of barriers, how they operate	Author 3 and another fully FICE-trained Consultant Anaesthetist
2	Focus groups (*n* = 3)	Preliminary findings validation via participants with multiple experience level and length	Author 1, Author 2	1.Conceptual understanding of the mechanism ‘vicious cycles’ in stopping POCUS-use, 2.Identify solutions to overcome those 3. Expand findings with multiple perspectives from practitioners	Intensivists and Anaesthetists with different length and level of experience in using FICE; Author 3
3	Interviews (*n* = 4)	Validate findings and reach data saturation	Author 2	1. Collected more practice-based examples applying theory 2. Data saturation reached	Author 1, Author 3
4	Echo-network workshops with expert panel (*n* = 2)	Findings validation	Author 1, Author 2	Confirming findings	Clinical leaders; Author 1 Author 2 Author 3

## Data Availability

No datasets were generated or analysed during the current study.

## Footnotes

1 The notion of Legitimate peripheral participation (LPP) describes how novices or learners of a profession become expert members of a professional community of practice; it analyses learning as a social phenomenon which requires that those who do not yet hold expert skill are able to participate legitimately in shared expert practices, and be supported by the expert community of practice in doing so. (Lave & Wenger, 1991)

2 An activity system (AS) is understood, by CHAT, as a collaborative, holistic system - such as a healthcare institution/department - that generates (professional) actions to address a collective purpose. This lens to professional learning highlights that the shared purpose(s) of the collective activity shape the possible actions that professionals within the system can take. In practice, practitioners’ actions in the AS are further shaped by its rules and norms, established division of labour and tools used by its members to do their work (Engeström & Pyörälä, 2021).

3 The difference-within-similarity (Hofmann, 2020) approach is an analytic method for systematically exploring through qualitative data how change happens in practice (or does not). It systematically seeks to identify and compare situations that are similar in some key conceptually and empirically relevant ways (e.g., situations with similar expert resources, or patients) in which different outcomes have emerged (support/no support; opportunity to scan/no opportunity), identifying features that may have contributed to that difference; it then takes those features as a new starting point and laterally seeks for more evidence of those features and examines their impact in other situations (Hofmann, 2024) to test if they act as a change mechanism.
